# Examining the Impact of Patient Demographics on Outreach Efforts for a Student-Run LGBTQIA + Free Clinic

**DOI:** 10.1007/s10900-025-01517-y

**Published:** 2025-11-13

**Authors:** Geoffrey Xue, Anthony Barnes, Gabriel Alexander Lee, Lucas Raphael Lee, Courtney DuBois Shihabuddin

**Affiliations:** 1 The Ohio State University College of Arts and Sciences, Columbus, OH USA; 2 The Ohio State University College of Medicine, Columbus, OH USA; 3 The Ohio State University College of Nursing, Columbus, OH USA; 4 The Columbus Free Clinic, Columbus, OH USA

**Keywords:** LGBTQIA + community, Sexual and gender minorities, Health disparities, Student-run clinics, Community outreach

## Abstract

The LGBTQIA + community faces unique health disparities that can lead to severe barriers to care. The Rainbow Clinic is a specialty clinic at the Columbus Free Clinic, a student-run free clinic affiliated with a large academic institution in the Midwest, that provides free primary and specialty care for uninsured and underinsured patients in Columbus, Ohio. Unfortunately, there is a paucity of literature depicting how to best reach these patient populations. This study sought to examine the outreach strategies for patient recruitment to the Rainbow Clinic with a retrospective chart review of past patients (*N* = 77). 44.2% of respondents discovered the clinic through social media. Age strongly and negatively correlated with appointment frequency (*r* = -0.665, *p* < 0.001), while income showed a moderate positive correlation (*r* = 0.464, *p* < 0.001). Preliminary data is a promising foundation for future research to improve recruitment of vulnerable populations. Correlations between age and social-media related appointments and income and social-media associated appointments can be helpful to address specific modes of outreach for target populations in the LGBTQIA + community.

## Introduction

The lesbian, gay, bisexual, transgender, intersex, and asexual+ (LGBTQIA+) community is often subject to discrimination and faces various forms of adversity, such as homophobia, social stigma, poverty, homelessness, and barriers to healthcare [1]. The injustices that the LGBTQIA + community face have dramatically impacted health disparities within the community. These disparities impact the community’s physical and mental health. To note, gay and bisexual men face increased prevalence of HIV (human immunodeficiency virus), and MSM (men who have sex with men) experience greater risk of STDs (sexually transmitted diseases) [2, 3]. Cancer rates in the LGBTQIA + community are also disproportionately high [4], especially in transgender individuals undergoing gender-affirming hormone therapy [5].

The mental health of LGBTQIA + community is also drastically affected, with one nationwide study finding 41% of transgender respondents attempting suicide, compared to 1.6% of the general population [6]. LGBTQIA + older adults face unique health and aging needs, including disabilities, mental health disorders, but show strong resilience [7]. The LGBTQIA + community is at an increased risk of abusing alcohol, tobacco, and other illicit drugs [8]. There are several reasons why these trends may exist, including socioeconomic gaps for the LGBTQIA + community, which is further exacerbated for the transgender community [9]. LGBTQIA + individuals were more likely to be uninsured [10, 11], report a higher prevalence of homelessness [12], and often have poor experiences with healthcare providers [13]. These circumstances taken together culminate in the concept of minority stress, in which chronic stress rooted in ongoing social challenges leads to poorer health outcomes in minority communities. In a ten-year systematic review, it was found that even with workplace policies promoting LGBTQIA + equality, dominant heteronormativity and micro-aggressions fostered recurring psychological impacts, such as increased rates of anxiety and depression [14]. However, sexual orientation and gender identity rarely work in isolation, often intersecting with other demographic factors to worsen concurrent mental health issues. For example, River et al. recorded that when members of the LGBTQIA + community attempted to access already limited mental health and substance abuse services, distinct hostile environments as a result of racial identity perpetuated feelings of invalidation, silence, and shame among those seeking such care [15].

Given these disparities, free clinics are critical to the LGBTQIA + community. Access to care is a pressing issue when there are complex personal and structural barriers for marginalized groups, including poor mental health, addiction, and language barriers [16]. When examining recruitment efforts from clinical trials and other community engagement strategies, ethnic and racial minorities have historically low participation, given mistrust of the medical system and cultural barriers put into place [17]. However, there is a lack of literature on targeting and representing the LGBTQIA + community in health-based research [18].

Nevertheless, LGBTQIA + individuals are more likely to have multiple social media accounts and to use social media more frequently compared to their non-LGBTQIA + counterparts [19], with 80% of LGBTQIA + individuals in one study engaging with LGBTQIA+-specific social media [20]. Social media plays a significant role in LGBTQIA + peer connections, identity management, and social support [21], and LGBTQIA + youth are more likely to have online friends than their non-LGBTQIA + peers [22]. Consequently, social marketing has become especially crucial for reaching the LGBTQIA + community, who depend more on digital interactions. This retrospective chart review examines how members of the LGBTQIA + community learn about a student-run free clinic, with a specific focus on identifying referral sources and evaluating the clinic’s outreach strategies.

### Description of the Rainbow Clinic

The Rainbow Clinic operates as a specialty clinic under an existing student-run free clinic system called the Columbus Free Clinic, associated with a tertiary academic Midwestern University medical center [23]. Rainbow Clinics were hosted in a medical center-associated primary care practice, and services provided overlapped with the established free clinic framework. Healthcare providers, medical students, nurse practitioner students, and undergraduate student volunteers, who often identify as part of the LGBTQIA + community or had previous experience with LGBTQIA + identifying patients, were trained to provide culturally-competent care with an emphasis on working with LGBTQIA + people. Online modules through the LGBTQIA + Health Education Center and peer-led in-person training were used to onboard new volunteers.

## Methodology

### Ethical Considerations

This retrospective chart review was determined to be exempt from full review by The Ohio State University College of Medicine Institutional Review Board (IRB), as it involved analysis of previously collected clinical data. No identifiable patient information was reviewed, and only included patients aged 18 years or older.

### Study Population

Since opening in April 2022, ten clinics have occurred between April 2023 and August 2024. The scope of the study was defined following the addition of a survey question asking participants how they learned about the clinic. Patient appointments were available between 5:30 pm-9:00 pm of each clinic night.

Ninety-two respondents completed the appointment request form to be seen at the Rainbow Clinic. For the purposes of data analysis, fifteen respondents were excluded due to failure to complete the survey, or submitting multiple requests during the study period. For patients with multiple survey responses, only first responses were included, and the remaining responses were excluded.

Seventy-seven respondents were included in the period of the study. Demographic information was organized into sexual orientation, gender identity, race/ethnicity, age, and income categories. Additional information was collected during the survey, including name, pronouns, zip code, telephone number, preferred language, and other demographic and health-related questions about their medical care. All demographics were self-reported. As shown, sexual orientation, gender identity, and income demographics varied greatly, while race/ethnicity and age demographics did not differ as greatly (Table 1). A plurality of the patients seen in the clinic were cis-gender, White, between the ages of 18–34, and had an annual income under $34,000, below the Ohio yearly median income of $59,890 [24] and the national median annual income of $65,470 [25]. For additional demographic information about the participant cohort, refer to Table 1.

**Table 1:** Self-reported demographics (Sexual orientation, gender identity, race/ethnicity, age, and income)

Demographic	Response	Frequency (*n*)
Sexual Orientation*	Bisexual	23
	Gay	14
	Pansexual	11
	Lesbian	9
	Heterosexual	8
	Queer	3
	Asexual	1
	Heteroflexible	1
	Does not know	3
	Other	1
	Do not wish to report	2
Gender Identity	Female	29
	Male	17
	Trans-male	5
	Trans-female	2
	Trans-masculine	1
	Non-binary	15
	Queer	6
	Do not wish to report	2
	Female	29
Race	White	38
	Black or African American	20
	Asian	3
	Mixed race	2
	Other	5
	Do not wish to report	9
Ethnicity	Hispanic/Latino	14
	Non-Hispanic/Latino	54
	Other	1
	Do not wish to report	8
Age	18–24	24
	25–34	36
	35–44	7
	45–54	6
	55–64	3
	65+	0
	Do not wish to report	1
Income	< 25k	33
	25-34k	17
	35-49k	7
	50-74k	8
	75-99k	1
	100-150k	1
	Do not wish to report	10

*1 patient excluded from the Sexual Orientation data based on their response as “Normal”

### Measures

The survey was accessed online via QR code or URL link. Walk-in patients were excluded from the study. The survey was disseminated in various ways (Table 2) and was written in English. Data was collected by retrospective review of the survey responses. Respondents self-reported answers to the question (“How did you hear about us?”) at the end of the survey. Two researchers coded responses into six categories: (1) word of mouth; (2) email or text; (3) social media; (4) referrals; (5) in-person outreach; and (6) web search. Word of mouth includes respondents who have heard about the clinic through friends, family, or other close relationships. Email or text includes respondents who may have heard about the clinic through email or text mailing lists. Referrals include respondents directly referred by community partners or other clinics. In-person outreach encapsulates respondents who heard about the clinic through community Pride festivals, tabling fairs, and other related activities.

**Table 2:** Summary of outreach strategies from the Rainbow Clinic

Community outreach strategy	Details
Produce effective materials	Design outreach materials including signage, social media posts, and email templates. Develop a relevant logo for the Rainbow Clinic brand. Create a QR code for patient appointment sign-up. Create a website dedicated to the clinic.
Engage with the LGBTQIA + events	Attend 4 + local Pride festivals annually. Attend LGBTQIA + community events. Attend LGBTQIA + focused conferences. Place flyers in libraries and public spaces. Create an email list for interested parties.
Connect with LGBTQIA + leaders and advocates	Engage with LGBTQIA + community meetings such as the Greater Columbus LGBTQIA + Health Coalition. Advocate for research focusing on LGBTQIA + needs. Identify LGBTQIA + owned businesses.
Partner with community organizations	Create a standard 30-minute presentation with Q&A for community partners. Obtain sexual health resources from community partners. Add Rainbow Clinic as a resource on partner websites. List Rainbow Clinic as a referral option.
Disseminate information via social media	Post materials twice before clinic dates on Instagram and Facebook at 2-weeks and 1-week before clinic date. Direct message partner organizations on Instagram once 2 weeks prior to clinic date. Email LGBTQIA + leaders and advocates twice 2-weeks and 1-week prior to clinic date. Keep appointment sign-up link on Instagram at all times.

### Analyses

A descriptive frequency table was used as a preliminary method of summarizing outreach methods for survey respondents. Additional separation based on predetermined age groups (i.e. 18–24, 25–34, 35–44, etc.) and income levels (< 25k, 25-34k, 35-49k, etc.) provided an initial visualization of referral methods across specific demographics. These groupings were selected to facilitate a comparison across different age and income segments.

To explore associations between demographic characteristics and referral patterns, Pearson’s correlation coefficient (*r*) was calculated to assess the relationship between age and income level (independent variables) and the frequency of social media as a reported referral source (dependent variable). Age and income level were treated as continuous variables and were ungrouped to reflect the accuracy of the data. The dependent variable was operationalized as the proportion of respondents who listed social media as their main method of identifying the clinic. Among the various outreach methods reported, social media was selected for further analysis due to its relatively high frequency in the descriptive data and relevance in existing literature, which highlights the prominent role of social media in healthcare engagement among younger LGBTQIA + individuals.

Analyses were performed using quantitative formulas in Microsoft Excel (version 2021, Redmond, Washington) and IBM SPSS Statistics (version 2024, IBM Corporation, Armonk, New York). P-values < 0.05 were set as statistically significant, and all p-values were two-sided.

## Results

Table 2 lists the comprehensive outreach strategies taken by clinic volunteers, shared to promote transparency and provide guidance for other student-run free clinics and community health settings. Table 3 lists the methods respondents heard about the clinic, with most of the respondents becoming aware of the clinic through social media platforms (43.9%). Other patients heard about the clinic through word of mouth (16.7%), including from friends and family, or a web search (13.6%). The lowest two methods were referrals (9.1%) and email or texts (6.1%), including the established mailing list.

**Table 3:** How respondents became aware of the Rainbow Clinic via appointment request form

	%	*n*
Word of mouth	16.9	13
Email or text	5.2	4
Social media	44.2	34
Referrals	7.8	6
Web search	13.0	10
In-person outreach	10.4	8
Do not wish to report	2.6	2

Table 4 lists how respondents became aware of the clinic separated by different age groups. In every age group, social media was the most common way respondents heard about the clinic, except in the 55–64 age group, where word of mouth was the most common. Other methods ranked more variably among age groups, with in-person outreach as the second highest category in the 18–24 age group, word of mouth and web search as the second highest tied categories in the 25–34 age group, in-person outreach and web search as the second highest tied categories in the 35–44 age group, email or text, referrals, and in-person outreach as second highest tied categories in the 45–54 age group, and email or text as the second highest category in the 55–64 age group.

**Table 4:** How respondents became aware of the Rainbow Clinic via appointment request form separated by age

Age Bracket	Word of mouth (%)	Email or text (%)	Social media (%)	Referrals (%)	Web search (%)	In-person outreach (%)
18–24	10.0	0	55.0	10.0	10.0	15.0
25–34	18.2	6.1	39.3	9.1	18.2	9.1
35–44	0	0	60.0	0	20.0	20.0
45–54	0	20.0	40.0	20.0	0	20.0
55–64	66.7	33.3	0	0	0	0

Table 5 lists the methods that respondents became aware of the clinic separated by different income levels. In respondents with an income level of under $25,000, $25,000-$34,000, and $50,000-$74,00, social media was the most common way respondents heard about the clinic. In the other income level groups, in-person outreach was the most common for the $35,000-$49,000 group, and word of mouth was the most common for the $75,000-$99,000 and $100,000-$150,000 groups.

**Table 5:** How respondents became aware of the Rainbow Clinic via appointment request form separated by income

Income	Word of mouth (%)	Email or text (%)	Social media (%)	Referrals (%)	Web search (%)	In-person outreach (%)
< 25k	25.0	7.1	28.6	10.7	21.4	7.1
25-34k	5.9	5.9	58.8	5.9	5.9	17.6
35-49k	0	0	40.0	0	0.0	60.0
50-74k	0	11.1	77.8	0	11.1	0
75-99k	100.0	0	0	0	0	0
100-150k	100.0	0	0	0	0	0

Pearson’s correlation coefficient (*r*) for the relationship between age and frequency of social media-related appointment requests (*r* = −0.665, p = < 0.001) indicates a strong negative correlation between the groups (Table 6). Pearson’s correlation coefficient (*r*) for the relationship between income level and frequency of social media-related appointment requests (*r* = 0.464, p = < 0.001) indicates a moderate positive correlation between the groups.

**Table 6:** Correlations between social-media related appointment requests, age, and income level

		Age	Income level
Frequency of Social Media-Related Appointment Requests	Pearson Correlation	− 0.665^**^	0.464^**^
	Sig. (2-tailed)	< 0.001	< 0.001
	N	66	61

**Correlation is significant at the 0.01 level (2-tailed)

## Discussion

Over 16 months, the Rainbow Clinic received seventy-seven unique patient appointment requests as a part of this study investigating how respondents learned about the clinic. Limited data is available on undergraduate students’ comprehensive, low-cost outreach efforts in a student-run free clinic, especially one that provides for the LGBTQIA + community. The LGBTQIA + community is a historically marginalized group and faces significant health disparities, contributing to and exacerbating avoidance in seeking healthcare [26]. The Rainbow Clinic provides an affirming, culturally-competent space for the LGBTQIA + community to access healthcare. The clinic sees multiple recurring patients, demonstrating the need for continuity of care and the presence of the clinic for the LGBTQIA + community. A new clinic was recently implemented within the Rainbow Clinic, establishing PrEP services for our patients.

Given the novel creation of the Rainbow Clinic, compiling a list of outreach efforts and subsequent patient data can demonstrate which efforts were most successful for the clinic. In this study, most respondents heard about the clinic through social media. A negative trend between age and frequency of social-media related appointments was observed, emphasizing that younger populations more often heard about the Rainbow Clinic through social media. Previous literature has established the importance of social media-based connections for youth and young adult LGBTQIA + individuals, further corroborating our data [21, 22]. Notably, higher income respondents more often heard about the Rainbow Clinic through social media. This pattern highlights the need to consider socioeconomic differences when designing inclusive and representative outreach strategies, especially when attempting to reach a larger pool of potential patients. To ensure equitable access, free clinics should diversify their outreach methods beyond social media. These methods can improve their ability to engage patients from vulnerable, underserved, and/or elder communities, who may rely less on social media.

### Limitations

The study has several limitations. The survey was written in English, so those unable to read English could have been deterred from signing up for an appointment, although digital translation services could have been used. Additionally, walk-in patients were excluded from the study, potentially representing a population not accounted for. Furthermore, sample size was limited, especially lacking older members of the LGBTQIA + community and gender diverse individuals. Future studies with greater sample size can be subject to additional analyses including the chi-square test. The study does not indicate a causal relationship between which methods were more effective for patient recruitment, and non-linear correlations or complex multivariate relationships were unable to be accounted for. Furthermore, the survey may be subject to self-report bias, which may not accurately capture how respondents heard about the clinic.

## Conclusion

This retrospective chart review offers a unique perspective on LGBTQ-focused community health settings, especially highlighting low-cost outreach efforts targeting vulnerable populations. Outreach efforts used in this study can be further expanded for the Rainbow Clinic and other student-run free clinics in the future. Based on the trend between age and frequency of social media-related appointment requests, younger individuals in the LGBTQIA + community may be more easily targeted through means of social media. In the context of free clinics or student-run free clinics, the specific outreach strategies outlined in this study, especially those via social media, may assist in patient recruitment for the younger individuals in the LGBTQIA + community. Given the rise of multiple forms of social media, more specific research on which form of social media is most effective for patient recruitment in free clinics can be of future interest. Additionally, in relation to income levels, those with a higher income were associated with higher frequency of social-media related appointment requests. Ultimately, supplementing social media outreach with additional outreach methods can help reach LGBTQIA + populations with limited online access. Future research should focus on conducting more in-depth analyses of the clinic’s outreach efforts to implement evidence-based practices in clinical approaches.

## Data Availability

The participants of this study did not give written consent for their data to be shared publicly, so due to the sensitive nature of the research supporting data is not available.
